# Network-targeted transcranial direct current stimulation of the hypothalamus appetite-control network: a feasibility study

**DOI:** 10.1038/s41598-024-61852-3

**Published:** 2024-05-18

**Authors:** Theresa Ester-Nacke, Katharina Berti, Ralf Veit, Corinna Dannecker, Ricardo Salvador, Giulio Ruffini, Martin Heni, Andreas L. Birkenfeld, Christian Plewnia, Hubert Preissl, Stephanie Kullmann

**Affiliations:** 1 Institute for Diabetes Research and Metabolic Diseases (IDM) of the Helmholtz Center Munich at the University of Tübingen, Tübingen, Germany; 2 Department of Internal Medicine, Division of Endocrinology, Diabetology and Nephrology, Eberhard Karls University Tübingen, Tübingen, Germany; 3 German Center of Diabetes Research (DZD), Tübingen, Germany; 4 Department of Psychiatry and Psychotherapy, German Center for Mental Health (DZPG), Neurophysiology and Interventional Neuropsychiatry, University Hospital Tübingen, Tübingen, Germany; 5 Department for Diagnostic Laboratory Medicine, Institute for Clinical Chemistry and Pathobiochemistry, Eberhard Karls University Tübingen, Tübingen, Germany; 6 Division of Endocrinology and Diabetology, Department of Internal Medicine 1, University Hospital Ulm, Ulm, Germany; 7 Neuroelectrics Barcelona, Barcelona, Spain

**Keywords:** Obesity, Cognitive control, Hypothalamus

## Abstract

The hypothalamus is the key regulator for energy homeostasis and is functionally connected to striatal and cortical regions vital for the inhibitory control of appetite. Hence, the ability to non-invasively modulate the hypothalamus network could open new ways for the treatment of metabolic diseases. Here, we tested a novel method for network-targeted transcranial direct current stimulation (net-tDCS) to influence the excitability of brain regions involved in the control of appetite. Based on the resting-state functional connectivity map of the hypothalamus, a 12-channel net-tDCS protocol was generated (Neuroelectrics Starstim system), which included anodal, cathodal and sham stimulation. Ten participants with overweight or obesity were enrolled in a sham-controlled, crossover study. During stimulation or sham control, participants completed a stop-signal task to measure inhibitory control. Overall, stimulation was well tolerated. Anodal net-tDCS resulted in faster stop signal reaction time (SSRT) compared to sham (*p* = 0.039) and cathodal net-tDCS (*p* = 0.042). Baseline functional connectivity of the target network correlated with SSRT after anodal compared to sham stimulation (*p* = 0.016). These preliminary data indicate that modulating hypothalamus functional network connectivity via net-tDCS may result in improved inhibitory control. Further studies need to evaluate the effects on eating behavior and metabolism.

## Introduction

Transcranial direct current stimulation (tDCS) represents a non-invasive brain stimulation (NIBS) method to modify cortical excitability in the brain^1^. For this purpose, tDCS uses a weak and constant direct current (DC) of about 1–2 mA, which is applied via electrodes through the scalp to the brain^2^ for a duration of commonly 20 min^3^, where it produces a weak electric field (E-field)^3^. The underlying principle of action is based on a subthreshold modulation of neuronal membrane potentials, leading to an alteration of the cortical excitability^4^. These changes in cortical excitability can be amplified or attenuated depending on the target area and current flow^5^. When stimulating the primary motor cortex, it was shown that the excitability is enhanced within the first 120 min after tDCS^6^. Transcranial direct current stimulation has already been applied in the treatment of various clinical conditions such as depression, anxiety and chronic pain^7^ and was shown to reduce seizure frequency in epilepsy^8^ as well as binge eating episodes^9^. Besides treatment and rehabilitation, tDCS can enhance the ability of working memory^10^ and cognitive control^11^. Although tDCS shows promising effects, meaningful clinical impact still needs to be confirmed for most conditions^12^.

So far, most of the tDCS studies targeted specific brain areas by bipolar tDCS, which usually includes two large sponge electrodes (one anode, one cathode)^2^. Thereby, the stimulating electrode is placed over the target region, while the reference electrode is located remotely^13^. More recently, it is possible to target brain regions more focally using high-definition (HD)-tDCS by applying multiple smaller electrodes. This has been reported to improve target intensity and focality^14^. By using multiple electrodes, it is also possible to stimulate brain networks, which is referred to as network-targeted tDCS (net-tDCS) stimulation.

Information on variation in network organization is primarily captured by resting-state functional connectivity (FC) using functional magnetic resonance imaging (fMRI)^15^. For instance, Fischer et al.^16^ showed that excitability of the left primary motor cortex (M1) and its associated resting-state network was more than twofold increased when using net-tDCS compared to bipolar tDCS targeting solely the M1. This is in line with a recent study where net-tDCS was able to induce greater FC during and after stimulation compared to bipolar tDCS when stimulating the sensorimotor network^17^. Results indicate the possibility of using a network-specific stimulation approach to activate numerous regions of a specified network, which could potentially lead to an overall more prominent modulatory effect^18^.

To influence eating behavior, previous tDCS studies have primarily focused on stimulating parts of the lateral prefrontal cortex, due to its fundamental role in inhibitory control^19^. Poor inhibitory control is thought to be involved in the development and maintenance of obesity^20^ and neuroimaging studies indicate that a diminished prefrontal cortex activity affects response inhibition^21^. Previous studies have therefore used predominantly bipolar tDCS to stimulate parts of the lateral prefrontal cortex, with the goal to increase inhibitory control^22^, reduce food intake and food craving (for review see^23^). Results are however not conclusive, with small moderating effects of tDCS on inhibitory control in single-session designs^24^. This could be because brain regions are continuously interacting and do not act in isolation^16,25^. Indeed, synchronized activation of the dlPFC and vmPFC has been shown to be necessary for successful dietary self-control^26,27^. Hence, it could be beneficial to stimulate an entire brain network vital for the inhibitory control of eating, rather than a single brain area.

The hypothalamus is crucial for whole body energy homeostasis and communicates with other regions in the brain and peripheral organs to control a wide range of neuroendocrine, metabolic and behavioral processes^28^. Resting-state FC studies revealed FC between the hypothalamus and regions important for motivation, reward and emotions as well as cognitive control^28–30^, particularly FC to striatal and bilateral medial and lateral prefrontal regions^31^. Obesity is associated with hyperactivity and higher FC in reward-related regions, as the striatum, and hypoactivity and lower FC in regions involved in cognitive control^32^. Moreover, hypothalamus resting-state FC to prefrontal and striatal regions is particular responsive to changes in satiety levels and hormonal modulations, an effect that is diminished in persons with obesity and insulin resistance^28,33^. Hence the hypothalamus network could be an ideal target to influence eating behavior and inhibitory control through its functional connections to striatal and prefrontal regions.

In the current pilot study, we aim to explore the effects of anodal and cathodal net-tDCS targeting the hypothalamus (i.e. appetite) network on inhibitory control in persons with overweight or obesity. We hypothesized that A) net-tDCS would be well tolerated and B) anodal stimulation would enhance inhibitory control during a Stop-Signal-Task (SST).

## Materials and methods

### Study population

In total, ten individuals with overweight or obesity (5 males and 5 females, age 45.70 ± 14.64 years, body mass index (BMI) 30.11 ± 3.39 kg/m^2^ reported as mean ± SD) were recruited via mail. Recruitment process is provided in Suppl. Figure 1. Exclusion criteria included severe pre-existing conditions, neurological or psychiatric disorders, frequent severe headaches and type 2 diabetes (T2D). Suppl. Table 1 provides information about the baseline characteristics of the participants.

### Experimental design and procedure

**Figure 1:**
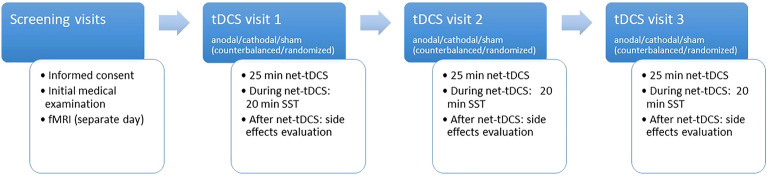
Experimental design. Screening was conducted prior net-tDCS visits. On the day of the screening visits, participants received an initial medical examination and a whole-brain resting-state fMRI was obtained on a separate day. On three separate visits (separated by 5–8 days), net-tDCS using three different protocols (anodal, cathodal, sham) took place in a pseudorandomized order. During net-tDCS, participants performed a 20 min SST. Subsequently after stimulation, participants filled out a questionnaire regarding side-effects. Abbreviations: fMRI, functional magnetic resonance imaging; SST, Stop-signal task.

The study was conducted as a double-blind, crossover, sham-controlled study comparing anodal and cathodal net-tDCS versus sham stimulation. The experimental design is shown in Fig. 1 (reporting checklist for tDCS studies see Suppl. Table 2). A block randomization procedure was used for the allocation of the participants. Participants underwent three identical sessions of anodal, cathodal, or sham net-tDCS, with a wash-out period of five to eight days following each session. On the tDCS visits, subjects arrived at 08:00 am after an overnight fast. To ensure a distraction-free environment, the stimulation took place in a separate quiet test room. Participants then underwent net-tDCS for 25 min and completed a 20 min SST to measure response inhibition. The investigator was in the same room but without being able to observe the task performance of the participant. At the end of each stimulation, a questionnaire evaluating possible adverse effects of the stimulation was administered. Prior to the tDCS-sessions, participants underwent a screening visit with resting-state fMRI, to assess resting-state FC of the hypothalamus network, and an initial medical examination to assess BMI, fasting glucose levels and peripheral insulin resistance.

### Tolerability of tDCS

Common side effects, based on previous literature^34^ (tingling, itching, pain and exhaustion), were assessed using a 100 mm Visual Analogue Scale (VAS) subsequently after stimulation. In addition, participants were asked on a 100 mm VAS, [“Overall, how uncomfortable was the stimulation for you?”]. Moreover, one line was left blank for additional side effects not listed above.

### Stop signal task

Response inhibition (i.e. inhibitory control) was assessed during net-tDCS, using the SST by CANTAB (Cambridge Neuropsychological Test Automated Battery)^35^. The primary outcome parameter is the stop signal reaction time (SSRT), which represents the duration of the inhibition process estimated from a mathematical model^36^.

For the task, participants touched a button on the left or right edge of a tablet computer screen using their left or right index finger as quickly as possible, every time an arrow in the center of the screen pointed in the corresponding direction. In 25% of the trials, an additional auditory signal (a beep) was presented, indicating the participant to withhold the response (response inhibition). In total, the task consists of a 16-item trial and five test blocks, each block consisting of 64 trials. The task uses a staircase design to adapt to the participants performance, achieving a 50% success rate for inhibition. The SSRT is calculated by a stochastic model that incorporates the average reaction time in runs without a stop signal, as well as the time interval between visual and auditory signal in which the subject is still able to withhold the reaction in 50% of cases^37^. This concept is considered adequate to measure inhibitory control, as the SSRT is assumed to equal the time before the action becomes ballistic and therefore one is no longer able to suppress it^38^.

### Network-targeted tDCS montage

**Figure 2:**
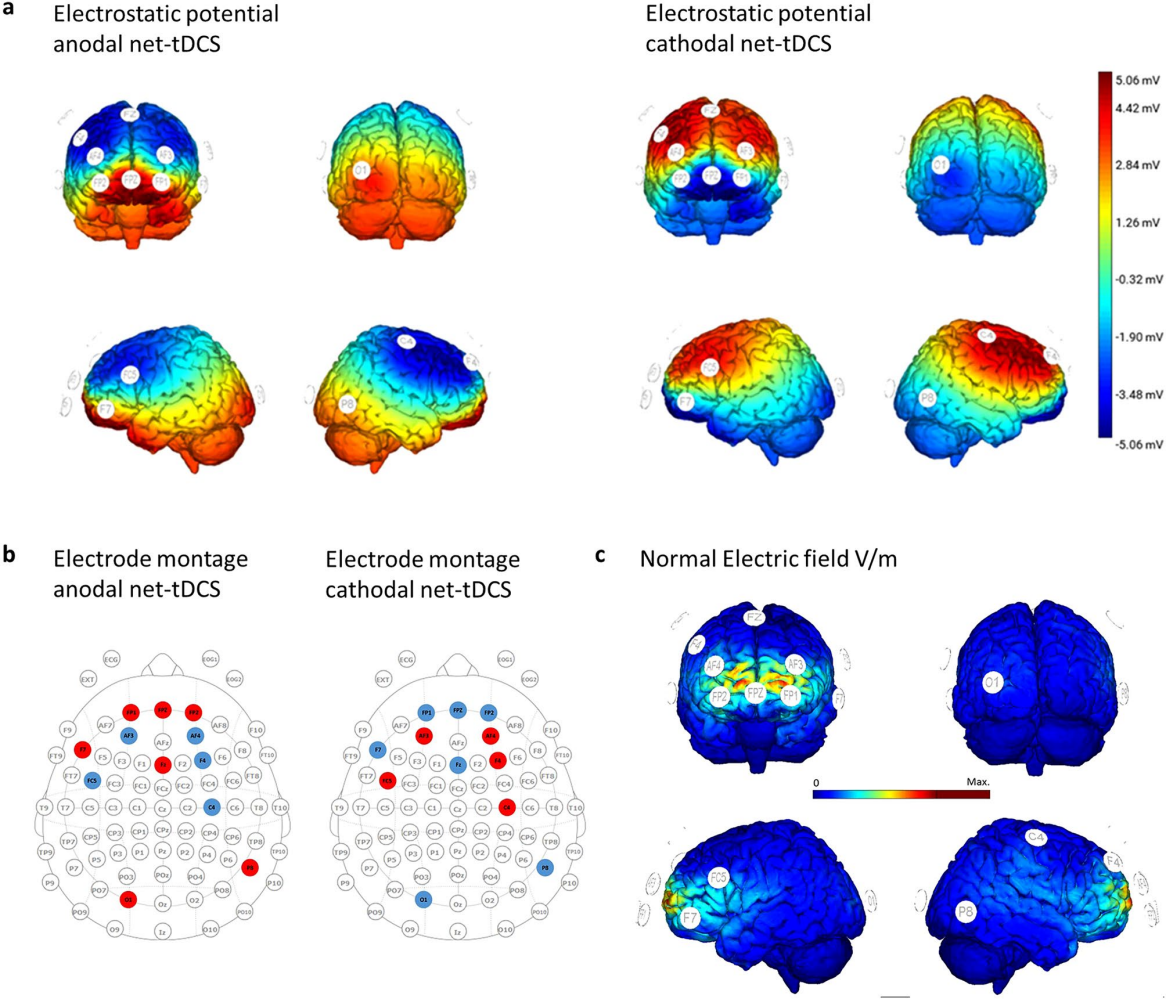
(**a**) Electrostatic potential of the anodal and cathodal net-tDCS. (**b**) Electrode montage for anodal and cathodal net-tDCS. Red dots indicate electrodes where current is conducted into the cortex, while blue dots show electrodes where current is dissipated. (**c**) Normal electric field (V/m) induced by a 12-electrode multichannel montage. Figures from Neuroelectrics Barcelona S.L.U.

Stimulation was delivered by a network-targeted multichannel tDCS device (Starstim^®^ 32, Neuroelectrics Barcelona S.L.U., Barcelona, Spain) with the corresponding NIC 2.0 software (NIC2 v2.0.11, https://www.neuroelectrics.com/resources/software). Twelve circular Ag/AgCl gelled electrodes were inserted into a neoprene cap with predefined positions (Headcap R, Neuroelectics Barcelona S.L.U., Barcelona, Spain) according to the 10–20 EEG international system (Fig. 2A–C and see Table 1 for electrode placement, current intensities and current density). To enhance current conductivity, the scalp at the electrode positions was gently rubbed with a cotton swab and an alcohol solution before stimulation. Active net-tDCS (anodal, cathodal) was delivered for 25 min, including a ramp-up of 15 s and a ramp-down of 60 s at the beginning and end of the stimulation. For sham tDCS, current was only delivered during ramp-up and ramp-down of the session. Impedance was kept below 10 kΩ during stimulation in order to minimize cutaneous sensations. Blinding was activated and maintained by using the NIC 2.0 Software (Neuroelectris Barcelona S.L.U., Barcelona, Spain). The anodal, cathodal and sham protocol was given a generic name from a third party, who then also activated the “double-blind” mode with a password requirement. Both the investigator and the participant were blind to the applied protocols.

**Table 1:** Electrode montage, applied current and current density of the net-tDCS for anodal and cathodal stimulation of the hypothalamus network.

Electrode placement	Anodal stimulation Applied current [μA]	Anodal stimulation Current density [mA/cm^2^]	Cathodal stimulation Applied current [μA]	Cathodal stimulation Current density [mA/cm^2^]
P8	251	0.08	− 251	− 0.08
F4	− 524	− 0.17	524	0.17
C4	− 353	− 0.11	353	0.11
FP2	345	0.11	− 345	− 0.11
FPZ	2000	0.64	− 2000	− 0.64
Fp1	342	0.11	− 342	− 0.11
AF3	− 1548	− 0.49	1548	0.49
Fz	357	0.11	− 357	− 0.11
AF4	− 1038	− 0.33	1038	0.33
O1	246	0.08	− 246	− 0.08
F7	459	0.15	− 459	− 0.15
FC5	− 537	− 0.17	537	0.17

The table shows the distribution of the applied current in μA. A max. total of 4 mA among the twelve electrodes was applied. Positive values indicate that the current is conducted into the cortex, negative values mean that the current is dissipated at this electrode. The electrode placement is based on the 10–20 international EEG system. For cathodal net-tDCS it should be noted that by inverting the current polarity of the electrodes, the effects of stimulation are inverted: areas that were inhibited before are now excited and vice-versa. This served as the rational for a montage to inhibit the excitability of the target network (cathodal net-tDCS).

Stimulation montage aimed to modulate the hypothalamus FC network. For this, the electrode positions and current per electrode were determined based on a computer algorithm (Stimweaver^™^ see^39^), resulting in a 12 channel optimization. The optimization was constrained to a total injected current of 4.0 mA and a maximum current at any given electrode of ± 2.0 mA. The E_n_-field calculations during the optimization was conducted on a template head model (Colin head model^40^). The target E_n_-field weights were defined based on the correlation strength obtained from the resting-state FC pattern of the hypothalamus from the Neurosynth website (https://neurosynth.org/locations/6_2_-10_6/). Stronger weights were given to areas with positive FC, as those had the stronger correlation coefficients (these are primarily frontal regions). Hence FC above 0.225 was assigned a weight of 10, everything below a FC of − 0.09 a weight of 4. For the range of the FC between − 0.09 and 0.225, the weights were scaled linearly. The resulting montage consisted of twelve π-cm^2^ size Ag/AgCl electrodes (Pistim, Neuroelectrics Barcelona S.L.U., Barcelona, Spain). In this optimization we focused on the E_n_-component of the E-field because it is thought to be the most relevant E-field orientation to excite/inhibit the pyramidal cells in the cortex: positive/negative E-field values (E-field directed into/out-off the cortical surface) lead to increases/decreases in the excitability. The optimization algorithm minimizes the least squares difference between the weighted target E_n_-field and the one induced by the montage.

The rationale for anodal net-tDCS followed the criteria presented in a previous study^16^, aiming to increase excitability in areas showing positive functional connectivity to the hypothalamus (i.e. regions showing positive correlation coefficients with hypothalamus) and inhibit excitability in those showing a negative functional connectivity. The cathodal net-tDCS aimed to inhibit excitability in areas showing positive functional connectivity and increase excitability in those showing a negative functional connectivity.

### Functional magnetic resonance imaging data acquisition

Whole brain functional fMRI data was obtained by using a 3.0 T scanner (Magnetom Prisma, Siemens Healthcare GmbH, Erlangen, Germany). Functional resting-state data were collected by using simultaneous multi–slice (SMS) sequence. The following sequence parameters were used: Acceleration factor = 4; TR = 1.18 s; TE = 34 ms; FOV = 205 mm2; flip angle 65°; voxel size 2.5 × 2.5 × 2.5 mm^3^; slice thickness 2.5 mm; images were acquired in an interleaved order. Each brain volume comprised 60 slices and each functional run contained 200 image volumes, resulting in a total scan time of 5 min. In addition, high-resolution T1 weighted anatomical images (MP2RAGE: 192 slices, matrix: 256 × 240, 1 × 1 × 1 mm^3^) of the brain were obtained.

### Resting-state fMRI Data processing

We used the Data Processing Assistant for Resting-State fMRI (DPARSF) (http://www.restfmri.net)^41^ to analyze the resting-state fMRI data. DPARSF is based on Statistical Parametric Mapping 12 (SPM12) (http://www.fil.ion.ucl.ac.uk/spm) and Resting-State fMRI Data Analysis Toolkit (REST, http://www.restfmri.net)^42^. The functional images were realigned and co-registered to the T1 structural image. The anatomical image was normalized to the Montreal Neurological Institute (MNI) template using DARTEL, and the resulting parameter file was used to normalize the functional images (voxel size: 2 × 2 × 2 mm^3^). Finally the normalized images were smoothed with a three-dimensional isotropic Gaussian kernel (FWHM: 4 mm). A temporal filter (0.01–0.08 Hz) was applied to reduce low frequency drifts and high frequency physiological noise. Nuisance regression was performed using white matter, CSF, and the six head motion parameters as covariates. No participant had head motion with more than 2.0 mm maximum displacement or 2.0° of any angular motion.

### Resting-state functional connectivity analyses

Resting-state FC maps of the 10 participants were obtained using a seed-based approach by computing resting-state FC between the hypothalamus and all voxels of the entire brain. We defined the hypothalamic region of interest using MNI coordinate hypothalamus x = 6, y = 2, z = − 10, including a 4 mm sphere. The resting-state FC maps were transferred to z values using Fisher’s transformation. Average FC values (z-transformed correlation coefficients) were extracted of the hypothalamus FC network. The extracted FC values were used to evaluate whether resting-state FC strength of the hypothalamus network predicted the net-tDCS induced changes in SSRT.

**Figure 3:**
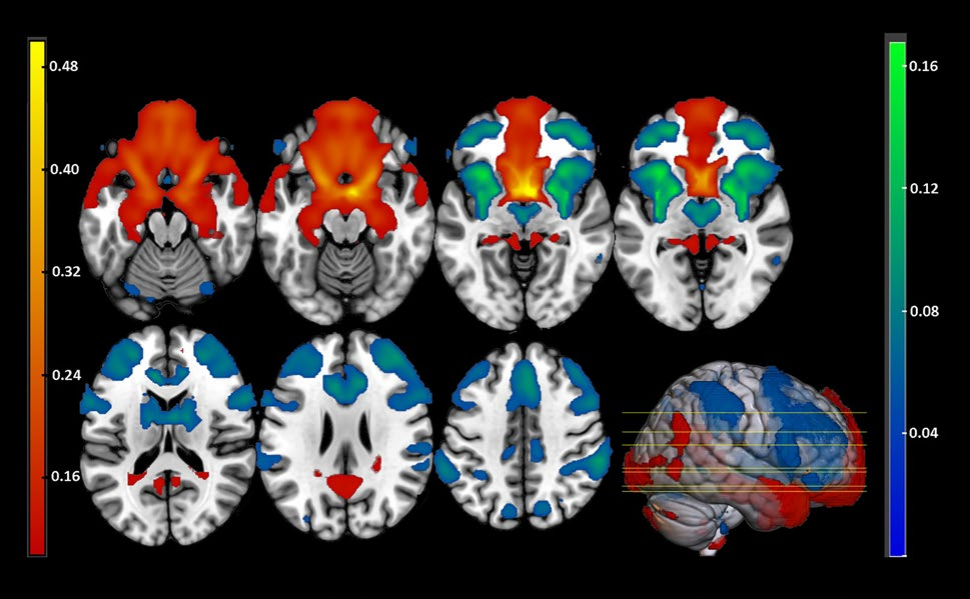
Hypothalamus FC map on a standardized T1 image computed using the Neurosynth website (https://neurosynth.org/locations/6_2_-10_6//) (thresholded at correlation coefficient r = − 0.005 and r = 0.005). Cortical areas with positive correlations to the hypothalamus were the bilateral ventromedial prefrontal cortex (PFC), orbital part of the inferior frontal gyrus (IFG), frontal pole, posterior cingulate cortex and hippocampus. Brain regions with negative correlations to the hypothalamus were the bilateral paracingulate cortex, dorsolateral PFC (including parts of the IFG), anterior insula, putamen, caudate, thalamus and orbitofrontal cortex. Yellow–red brain regions indicate positive correlations to the hypothalamus; blue-green brain regions indicate negative correlations to the hypothalamus.

Regions of the hypothalamus FC network were defined based on the resting-state FC pattern of the hypothalamus (https://neurosynth.org/locations/6_2_-10_6/) and separated into a map with fisher z-transformed positive FC coefficient values (threshold r > 0.005) and negative FC coefficients (threshold r < − 0.005). The corresponding map is depicted in Fig. 3 for illustration purpose. The two maps were further binarized, combined and used as a mask to extract the FC values of the 10 participants.

### Statistical analysis

Data is presented as mean and standard error of the mean (SEM) unless otherwise stated. For non-parametric data, we reported the median and interquartile range (IQR). Statistical analysis were performed using the program R (R version 4.3.3 (2024-02-29 ucrt), https://cran.r-project.org/bin/windows/base/). Statistical significance was set at *p* ≤ 0.05. A normal distribution of the parameters used was assessed by Shapiro–Wilk’s test. When data was not normally distributed, parameters were log-transformed prior analysis or non-parametrical analyses were conducted.

Net-tDCS effects on inhibitory control were analyzed using the SSRT of the SST by means of a Linear Mixed Effects Model (LMER) using the *lmer* function of the *lme4* package in R. As random effect we included a random intercept for each participant in order to account for differences at baseline between individuals. Stimtype (sham, anodal and cathodal net-tDCS), age and visit (visit 1, visit 2, visit 3) were included as fixed effects. Sham and visit 1 were defined as reference category in the model. Effect size was evaluated using the *effectsize* function of the *effectsize* package in R which supports *lmer* objects. Additionally, in an exploratory analysis, we compared the effects of anodal vs. cathodal net-tDCS using the same *lmer* function and framework. To associate changes in SSRT with baseline resting-state FC exploratory partial correlation analysis was used by *pcor.test* function of the *ppcor* package in R. Age was used as a covariate. A Friedman-Test was used to evaluate side effects of the stimulation.

### Ethics approval and consent to participate

The study protocol was approved by the ethics committee of the University Hospital Tübingen prior study start (project number: 243/2019BO1). Data acquisition was performed at the University Hospital of Tübingen between November 2019 and March 2020. All participants provided written informed consent prior study enrollment. All methods were carried out in accordance with relevant guidelines and regulations.

## Results

### Tolerability of net-tDCS

Both, active and sham net-tDCS were well tolerated and no participant discontinued the study due to side effects. Subsequently after stimulation, participants completed a VAS where the most common side effects were listed. Table 2 summarizes the mean values of the side effects across all stimulation protocols. There were no significant difference in experienced levels of discomfort between stimulation protocols (tingling χ^2^(2) = 0.171, *p* = 0.918; itching χ^2^(2) = 1.152, *p* = 0.562; pain χ^2^(2) = 0.703, *p* = 0.704; exhaustion χ^2^(2) = 3.813, *p* = 0.149; overall impression (“Overall, how uncomfortable was the stimulation for you?”) χ^2^(2) = 3.211, *p* = 0.201). In total, four individuals reported further side effects in addition to those already included on the questionnaire. 1) slight burning sensation at the beginning (1 × anodal, 1 × sham), feeling of warmth (1 × anodal, 1 × cathodal, 2 × sham) and a feeling of pressure or tension (1 × cathodal, 1 × sham). No severe adverse events occurred during the study.

**Table 2:** Subjective side effects.

	Anodal (n = 10)	Range (Min–Max)	Cathodal (n = 10)	Range (Min–Max)	Sham (n = 10)	Range (Min–Max)
Tingling	3.1 ± 5.8	0–8.7	5.1 ± 6.9	0–9.7	3.9 ± 5.6	0–7.7
Itching	0.4 ± 4.8	0–8.7	0.9 ± 6.9	0–8.3	0.2 ± 3.4	0–7.5
Pain	0.3 ± 0.9	0–8.8	0.3 ± 1.6	0.1–8.3	0.5 ± 1.0	0–8.5
Exhaustion	0.1 ± 0.8	0–1.5	0.4 ± 0.8	0–4.9	0.4 ± 0.9	0–5.2
Overall impression	4.1 ± 2.3	0–7.3	4.7 ± 2.1	0.3–6.0	4.4 ± 2.8	0.3–5.9

Subjective side effect measures of the net-tDCS for all three stimulation conditions reported by the participants on a 0–100 VAS. Data are Median ± IQR.

### Response inhibition during SST

**Figure 4:**
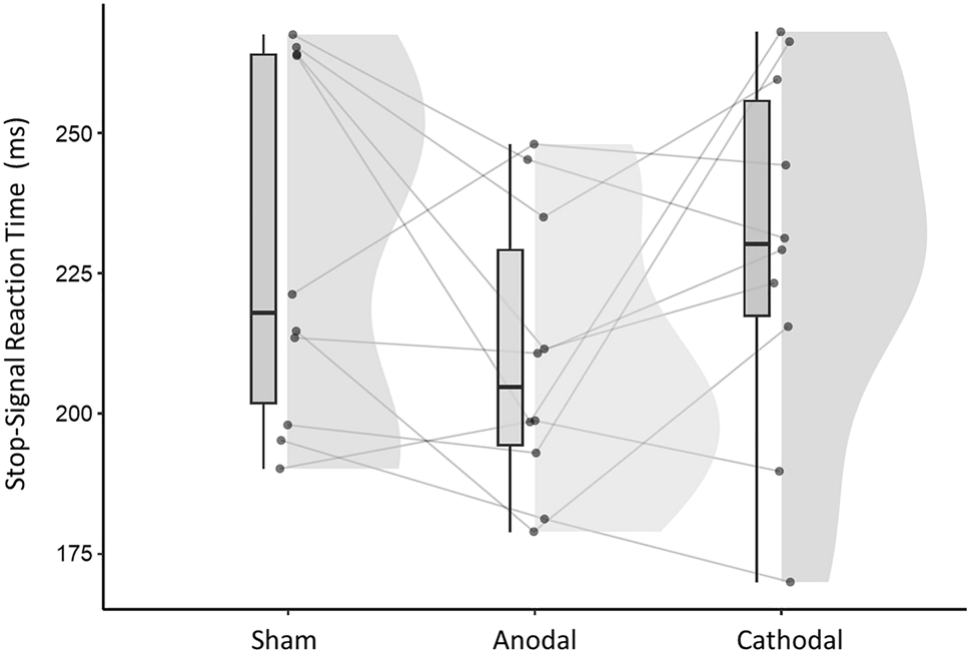
Raincloud plots of the raw SSRT. Each dot represents one participant in each condition (sham, anodal, cathodal net-tDCS). Box plots display the median and upper/lower quartiles for each stimulation condition and the histograms represent the data distribution. *N* = 10.

**Figure 5:**
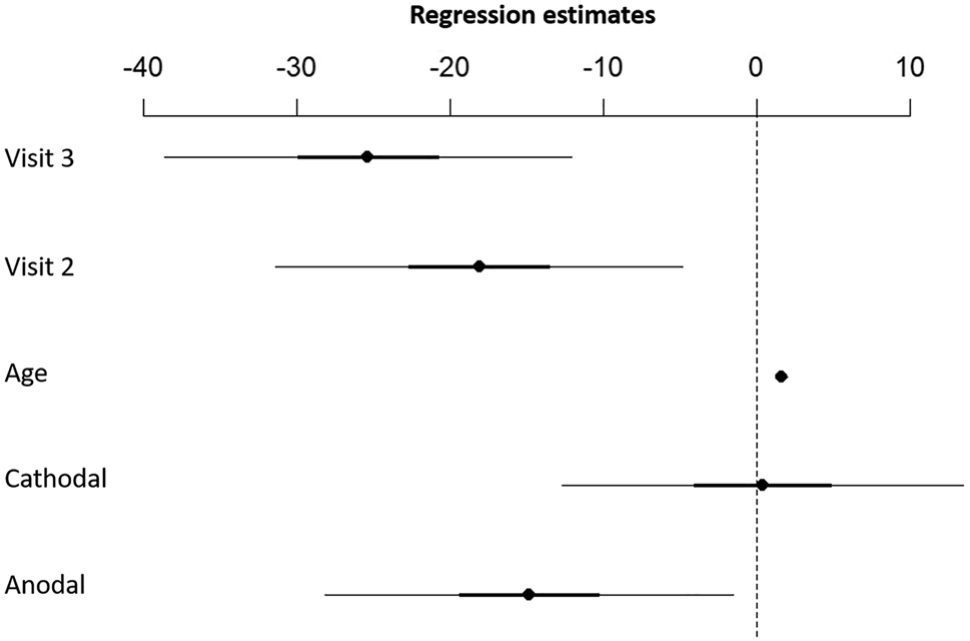
Effect of active net-tDCS on SSRT compared to sham stimulation. Plot shows regression estimates with 95% CI. Visits 2 and 3 showed shorter SSRT values compared to visit 1. Compared to sham, anodal net-tDCS resulted in shorter SSRT values. There was no effect of cathodal net-tDCS compared to sham stimulation. Age is a significant predictor indicating that SSRT values are higher with increasing age. *N* = 10.

The SST evaluates impulse control and response inhibition^35^. The primary outcome measure is the SSRT, which estimates the time a participant needs to successfully inhibit a planned response. The mean SSRT across the three stimulation protocols was as follows: anodal tDCS (210.1 ± 25.1 ms); cathodal stimulation (229.7 ± 32.2 ms); sham tDCS (229.3 ± 32.2 ms). Figure 4 depicts the raw values of the SSRT during all three stimulation conditions. We found a significant difference between anodal and sham net-tDCS (Estimate− 14.88, CI [− 28.93 –− 0.83], *p* = 0.039) with a medium effect size (std. coef. 0.48), indicating shorter SSRT during the anodal compared to sham. This indicates better inhibitory control during anodal than sham stimulation. There was no difference in the SSRT during the cathodal stimulation compared to sham (Estimate 0.36, CI [− 13.48–14.19], *p* = 0.958). There was a main effect of visit (visit 1 vs. visit 2 (Estimate − 18.14, CI [− 32.19–− 4.09], *p* = 0.014); visit 1 vs. visit 3 (Estimate − 25.41, CI [− 39.46–− 11.36], *p* = 0.001)) with longer SSRT during visit 1. Age was a significant predictor (Estimate 1.57, CI [1.17–1.98], *p* < 0.001) with longer SSRT with increasing age. See Fig. 5 for regression estimates of the model. Moreover, exploratory analysis showed significant higher SSRT during cathodal compared to anodal stimulation (Estimate 15.97, CI [0.71–31.22], *p* = 0.042).

### Correlation between resting-state hypothalamic FC and tDCS-induced changes in SSRT

**Figure 6:**
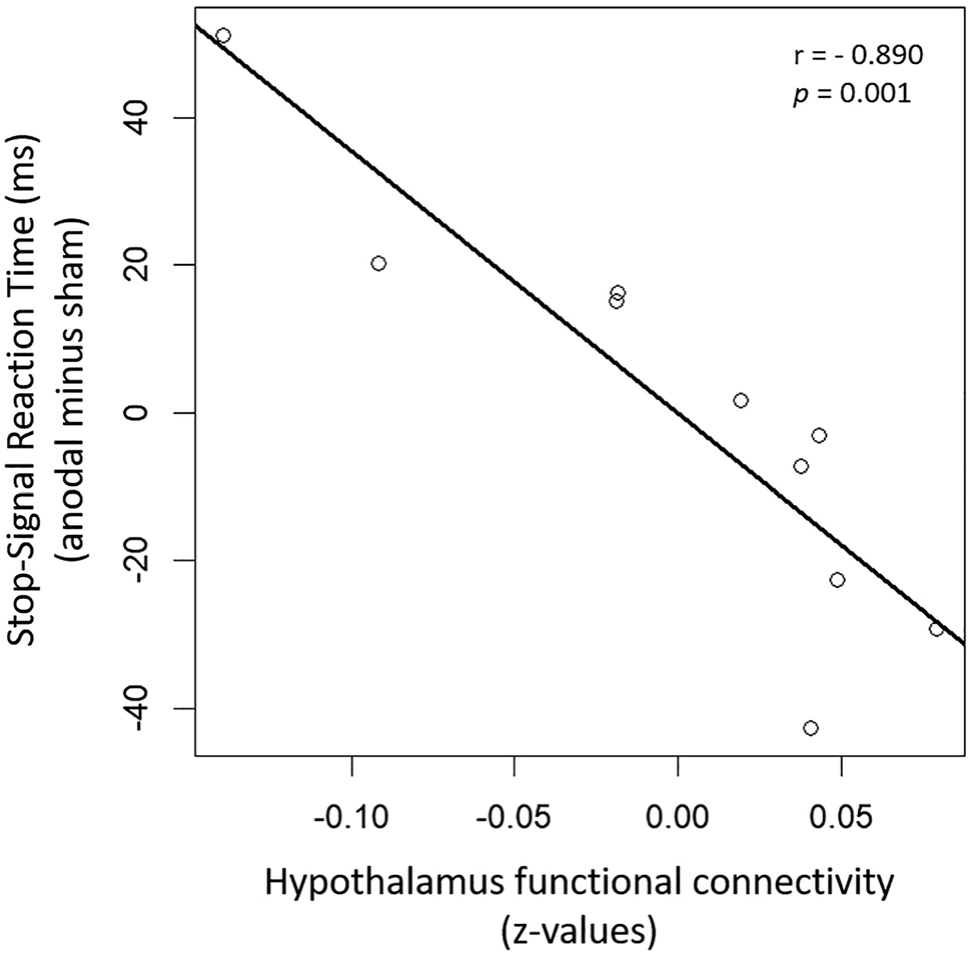
Partial correlation (corrected for age) between the hypothalamus resting-state FC at baseline and the changes in the SSRT after anodal stimulation compared to sham. Participants with higher hypothalamus FC show a stronger reduction in SSRT in response to anodal net-tDCS, which indicates better inhibitory control.

We evaluated whether resting-state FC of the hypothalamus network prior to stimulation correlated with net-tDCS induced changes in SSRT. Hypothalamic resting-state FC significantly correlated with changes in SSRT after anodal stimulation (compared to sham) (r = − 0.890, *p* = 0.001 adj. for age) (Fig. 6). No correlation was observed with SSRT after cathodal stimulation (r = − 0.496, *p* = 0.174 adj. for age). Hence, participants with higher hypothalamus resting-state FC prior to stimulation showed better cognitive performance (i.e. response inhibition) during SST in response to anodal compared to sham stimulation.

## Discussion

The present study evaluated the effects of net-tDCS targeting the hypothalamus appetite control network in a sham-controlled crossover experiment in persons with overweight or obesity. The network included regions functionally connected to the hypothalamus, including parts of the frontal cortex, posterior cingulate cortex, striatum, insula and hippocampus. The study focused on response inhibition using a novel approach to stimulate brain networks^16,17^. Specifically, the trial investigated the safety, feasibility and efficacy of a net-tDCS approach optimized to stimulate the appetite network, based on hypothalamus resting-state FC. Network-stimulation was well tolerated. Preliminary results suggest increased inhibitory control during anodal net-tDCS compared to sham and cathodal stimulation. Moreover, hypothalamic resting-state FC was predictive for the enhanced cognitive performance in the anodal condition. However, due to the small sample size of 10 individuals, results should be carefully interpreted and this novel approach requires further validation. This limitation must be taken into account when transmitting these results to larger populations, as the small number of subjects increases the risk of Type I and Type II errors. Future research should therefore prioritize a larger sample size in order to improve the reliability of the data and to better support the validity of the observed effects. A recent study evaluating the tolerability and blinding of small Ag/AgCl electrodes found the stimulation to be generally well tolerated and showed no safety-related adverse events^43^. Here, we used twelve π-cm^2^ size Ag/AgCl electrodes in an electrode placement according to the 10–20 EEG international system. All three stimulation protocols were well tolerated with only minor side effects and no differences were observed between sham versus anodal or cathodal net-tDCS. For multichannel stimulation, usually higher current intensities are used compared to bipolar tDCS^17^. The present study stimulated with a total injected current of 4 mA, whereas most bipolar tDCS studies apply 2 mA. A study evaluating the tolerability of 2 and 3 mA in a multichannel tDCS setting showed no serious adverse effects and tolerability did not differ between current intensities^44^. In addition, a recent study compared side effects of net-tDCS (4 mA) and bipolar tDCS (2 mA) targeted over the M1 and did not find differences between groups nor between active and sham stimulation regarding subjective sensations^17^.

Higher activity in frontal brain regions are associated with higher levels of dietary restraint^45^ and self-control in response to food^46,47^. Accordingly, individuals with obesity show diminished prefrontal cortex activity, which affects inhibitory control and the regulation of body weight^21,24^. Modulation of prefrontal cortex activity with tDCS has been used to enhance inhibitory control^24,48,49^. Individualized electrode montage over the dlPFC has been discussed to improve neuropsychological outcomes^50^, but no study has investigated network based tDCS and its effects on response inhibition so far. There is, however, first evidence showing increased resting-state FC within the sensorimotor network after net-tDCS compared to bipolar tDCS^17^. However, there are limited studies available that examined the outcome of net-tDCS on behavior. Gregoret et al.^51^ evaluated net-tDCS of the motor network on pain perception and electro-cortical response to pain. While no differences were observed on pain perception, net-tDCS was effective in modulating the neural response to pain^51^. Moreover, there is evidence that simultaneous stimulation of multiple brain areas increases inhibitory control. A multitarget HD-tDCS study concurrently targeted the right IFG and the pre-supplementary motor area (pre-SMA) and stimulated these brain regions individually for comparison. Results indicate that the combined stimulation of both regions led to improved inhibitory control, while no such improvement was observed when stimulating one single brain region^52^. Here, we tested the impact of net-tDCS optimized to modulate regions important for appetite control, which included parts of the dlPFC, vmPFC, IFG as well as limbic regions important for reward processes and decision making. We showed that anodal net-tDCS was able to enhance response inhibition compared to sham and cathodal net-tDCS. Further studies using net-tDCS are needed to evaluate whether the possible improvements result in enhanced cognitive control of eating.

A functional network is determined by positive as well as negative functional connections (i.e. correlations). The hypothalamus network showed positive correlations with parts of the ventromedial PFC, posterior cingulate cortex, and hippocampus. Negative correlations with the hypothalamus are found with parts of the dlPFC, insula and striatal regions. These negative correlations are also referred to as anti-correlations, which play a crucial role in the functional organization of the brain^53^ and have been found to be influenced by body weight^54^. In the current study, we took positive and negative FC into account in the development of the stimulation protocol. If and how these FC patterns were influenced by the current net-tDCS protocols still needs to be determined in future fMRI studies. There is evidence, however, that FC can be influenced by tDCS, which is related to improved response inhibition^49,55^. While these studies did not evaluate eating behavior, fMRI studies revealed that FC between the dlPFC and the vmPFC is linked to food-related impulse control^56^ and persons with obesity can learn to increase FC between these regions using neurofeedback training (i.e. a cognitive regulation skill)^57^.

In the current study, we demonstrated that hypothalamus resting-state FC at baseline predicted improved cognitive performance during anodal net-tDCS stimulation. There is evidence that resting-state FC is predictive for individual differences in cognitive performance as well as cognitive impairment^58,59^. Hence, alterations of brain networks and network-to-network interactions have been proposed as potential biomarkers in neurological and psychiatric conditions^60^. In persons with obesity, higher hypothalamic resting-state FC has been identified with the insula and striatum^28,61^ and lower FC in regions associated with cognition^32^. Moreover, hypothalamus resting-state FC to frontal areas were shown to be sensitive to changes to peripheral hormones^28,62,63^ and even predictive for weight loss^54^. In response to substantial weight-loss after bariatric surgery, hypothalamic FC has been shown to normalize with comparable FC patterns as in normal weight controls. Furthermore, dysfunctional hypothalamus FC has been documented in neurological and psychiatric diseases^28^. Hence, we postulate that the hypothalamus network could be an ideal target to influence physiological and psychological processes.

Overall, the present trial contributes to the understanding of initial resting-state FC as a possible predictor for tDCS related effects. Whether the current net-tDCS protocol in fact modulates hypothalamus FC still needs to be determined in future studies.

## Limitations and further directions

This is a pilot study and further investigations in larger sample sizes are necessary to evaluate the effects of net-tDCS targeting the hypothalamus network on eating behavior. In addition, it is important to acknowledge that no fMRI measurements were performed immediately after stimulation. Hence no conclusion can be drawn as to whether the implemented tDCS protocols resulted in changes in functional activity within the hypothalamus network. The present study used a net-tDCS paradigm, based on fixed electrode positions of the 10–20 international EEG system. Due to this standard configuration, individual anatomical differences were not taken into account, thus for some individuals we may not have targeted the optimal stimulation points. Future studies should therefore investigate the possibility of personalized electrode arrangements, for instance based on their unique brain anatomy and fMRI network dynamics.

## Conclusion

Overall, the present study adds novel findings to the growing body of research in the field of multifocal tDCS solutions. Here, we show that anodal net-tDCS based on resting-state fMRI connectivity patterns of the hypothalamus is well tolerated and able to enhance inhibitory control in individuals with overweight or obesity. Whether anodal net-tDCS of the hypothalamus appetite network can improve inhibitory control of eating behavior, reduce food craving or ultimately facilitate healthier food choices needs to be investigated in future studies.

### Supplementary Information

Supplementary Information.

## Data Availability

The data that support the findings of this study are available on request from the corresponding author, [TEN].
