# Supplementary material for: Network-targeted transcranial direct current stimulation of the hypothalamus appetite-control network: a feasibility study
Source: Sci Rep. 2024 May 18;14:11341. doi: 10.1038/s41598-024-61852-3 (PMC11102513; doi:10.1038/s41598-024-61852-3)
Supplement: Supplementary file 1 — Supplementary Information. [file 41598_2024_61852_MOESM1_ESM.docx]

**Supplementary information**

**Network-targeted transcranial direct current stimulation of the hypothalamus appetite-control network: A feasibility study**

Theresa Ester-Nacke^1,2,3†*^, Katharina Berti^1,2,3†^, Ralf Veit^1,2,3^, Corinna Dannecker^1,2,3^, Ricardo Salvador^7^, Giulio Ruffini^7^, Martin Heni^1,2,3,5,6^, Andreas L. Birkenfeld^1,2,3^, Christian Plewnia^4^, Hubert Preissl^1,2,3^, Stephanie Kullmann^1,2,3^

^1^Institute for Diabetes Research and Metabolic Diseases (IDM) of the Helmholtz Center Munich at the University of Tübingen, Tübingen, Germany.

^2^Department of Internal Medicine, Division of Endocrinology, Diabetology and Nephrology, Eberhard Karls University Tübingen, Tübingen, Germany.

^3^German Center of Diabetes Research (DZD), Tübingen, Germany.

^4^Department of Psychiatry and Psychotherapy, Neurophysiology & Interventional Neuropsychiatry, University Hospital Tübingen, Tübingen, Germany and German Center for Mental Health (DZPG).

^5^Institute for Clinical Chemistry and Pathobiochemistry, Department for Diagnostic Laboratory Medicine, Eberhard Karls University Tübingen, Tübingen, Germany.

^6^Division of Endocrinology and Diabetology, Department of Internal Medicine 1, University Hospital Ulm, Ulm, Germany.

^7^Neuroelectrics Barcelona, Barcelona, Spain.

^†^authors contributed equally

*Corresponding author:

Theresa Ester-Nacke – Institute for Diabetes Research and Metabolic Diseases (IDM) of the Helmholtz Center Munich at the University of Tübingen, Otfried-Müller Str. 47, 72076 Tübingen, Germany

e-mail: [Theresa.ester@med.uni-tuebingen.de](mailto:Theresa.ester@med.uni-tuebingen.de)

**Suppl. Figure 1.** Recruitment process.


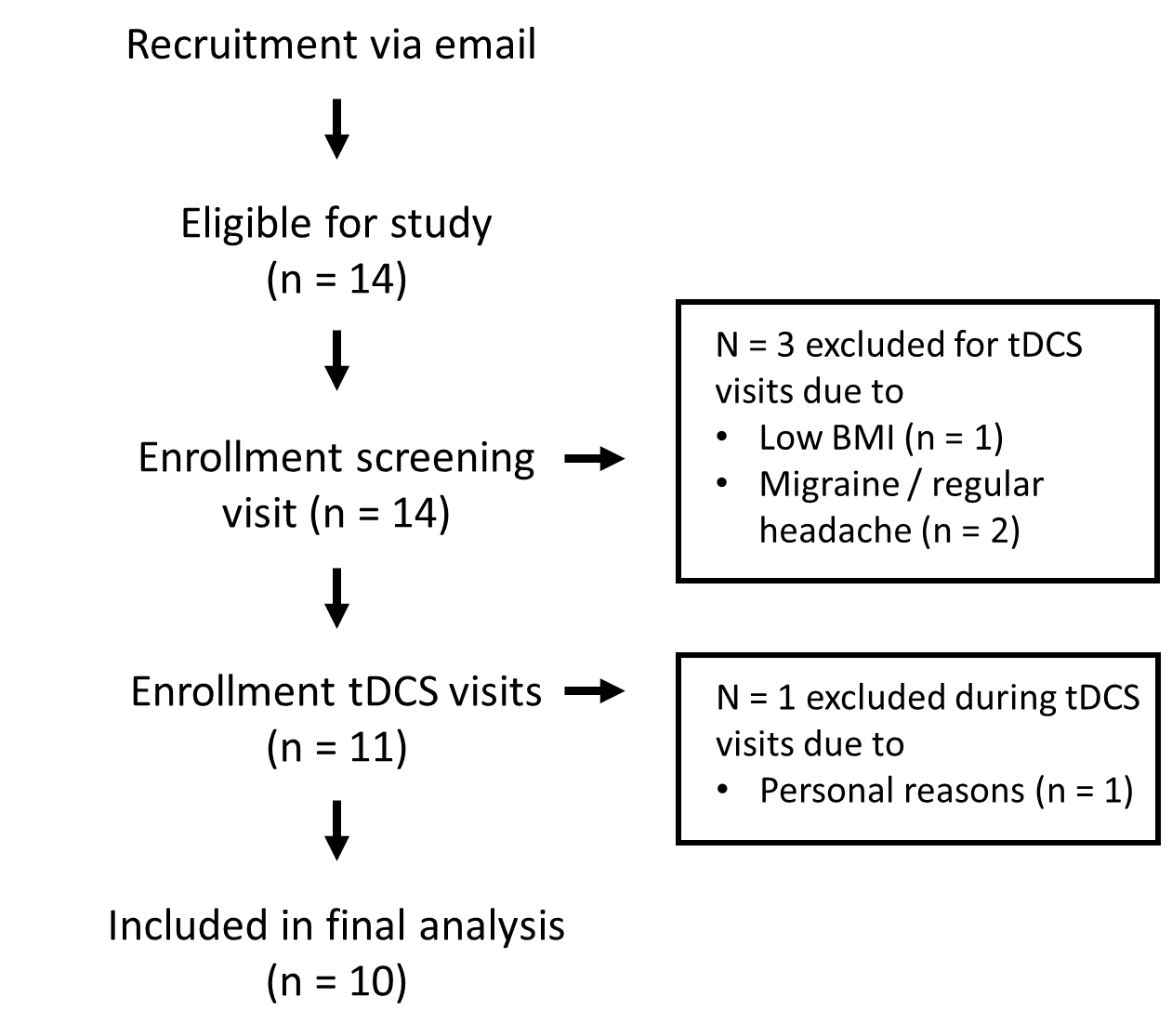


**Suppl. Table 1** Study population baseline characteristics.

| ***N= 10 (5 females)*** | ***Mean ± SEM*** | ***Range***  ***Min-Max*** |
| --- | --- | --- |
| ***Age (years)*** | 45.7 ± 4.6 | 23 - 60 |
| ***BMI (kg/m^2^)*** | 30.11 ± 1.07 | 25.2 – 37.0 |
| ***Fasting blood glucose level (mmol/l)*** | 5.53 ± 0.22 | 4.67 – 6.61 |
| ***HOMA-IR*** | 3.35 ± 0.42 | 1.92 ± 5.89 |

The table shows the anthropometric and metabolic data of the participants at baseline. Data is summarized as Mean ± SEM. The range of values (minimum to maximum) is given as well. Abbreviations: BMI, body mass index; HOMA-IR, Homeostasis Model Assessment of Insulin Resistance.

**Suppl. Table 2** Reporting checklist for tDCS studies based on Buch et al. [1]. Variant appropriate to our study are marked with an “X”.

| ***Experimental Design Factors:*** | | | |
| --- | --- | --- | --- |
| ***Controls used*** | □ None | X Sham | □ Active |
| ***Blinding used*** | □ None | □ Single | X Double |
| ***Hypothesis statement*** | X Yes | □ No |  |
| ***If Hypothesis-based:*** |  |  |  |
| ***Power-analysis statement*** | □ Yes | X No |  |
| ***Pre-registration*** | □ Yes | X No |  |
| ***Exploratory-based*** | X Yes | □ No |  |
| ***Sample-size estimation (i.e. – power analysis)*** | □ Yes | X No |  |
|  |  |  |  |
| ***Participant Factors:*** | Reported? | Controlled? |  |
| ***Number of subjects*** | X | □ |  |
| ***Age of subjects*** | X | □ |  |
| ***Gender of subjects*** | X | □ |  |
| ***Handedness of subjects*** | □ | □ |  |
| ***Subjects prescribed medication*** | □ | X |  |
| ***Use of CNS active drugs (e.g. anti-convulsants)*** | □ | X |  |
| ***Neuropsychological evaluation*** | □ | X |  |
| ***Any medical conditions*** | □ | X |  |
| ***History of specific repetitive motor activity*** | □ | □ |  |
| ***Years of Education completed*** | □ | X |  |
|  |  |  |  |
| ***Stimulation Factors:*** | Reported? | Controlled? |  |
| ***Scalp position of tDCS electrodes*** | X | □ |  |
| ***MRI-based localization of tDCS electrodes*** | □ | □ |  |
| ***Electrode type (size and geometry)*** | X | □ |  |
| ***Current density of applied stimulation*** | X | □ |  |
| ***Type of stimulator used (e.g. brand)*** | X | □ |  |
| ***Stimulation intensity*** | X | □ |  |
| ***Stimulation ramp time*** | X | □ |  |
| ***Stimulation duration*** | X | □ |  |
| ***Number of Sessions*** | X | □ |  |
| ***If Multiple Sessions:*** |  |  |  |
| ***Time interval between sessions*** | X | □ |  |
| ***Subject attention (level of arousal) during testing*** | □ | X |  |
| ***Subject activities during stimulation*** | X | □ |  |
| ***tDCS-induced sensations (i.e. – itching, pain, heat, pinching, burning)*** | X | □ |  |
|  |  |  |  |
| ***Analysis & Statistics factors:*** |  |  |  |
| ***Effect-size(s) reported*** | X Yes | □ No |  |
| ***Raw data uploaded to publicly accessible data repository*** | □ Yes | X No |  |
| ***Analyzed data uploaded to publicly accessible data repository*** | □ Yes | X No |  |
| ***Full analysis protocol including custom scripts uploaded to publicly accessible data repository*** | □ Yes | X No |  |

**References**

1 Buch ER, Santarnecchi E, Antal A *et al.* Effects of tDCS on motor learning and memory formation: A consensus and critical position paper. *Clin Neurophysiol* 2017;**128**:589–603.
